# Seasonal range fidelity of a megaherbivore in response to environmental change

**DOI:** 10.1038/s41598-022-25334-8

**Published:** 2022-12-22

**Authors:** Rhea Burton-Roberts, Line S. Cordes, Rob Slotow, Abi Tamim Vanak, Maria Thaker, Navashni Govender, Graeme Shannon

**Affiliations:** 1 School of Natural Sciences, Bangor University, Bangor, Gwynedd UK; 2 School of Ocean Sciences, Bangor University, Bangor, Gwynedd UK; 3 School of Life Sciences, University of KwaZulu-Natal, Pietermaritzburg, South Africa; 4 Centre for Biodiversity and Conservation, Ashoka Trust for Research in Ecology and the Environment, Bangalore, India; 5 Centre for Ecological Sciences, Indian Institute of Science, Bangalore, India; 6 Conservation Management, Kruger National Park, South African National Parks, Private Bag X402, Skukuza, 1350 South Africa; 7 School of Natural Resource Management, Nelson Mandela University, Private Bag X6531, George, 6530 South Africa

**Keywords:** Behavioural ecology, Biodiversity, Conservation biology, Ecology, Zoology

## Abstract

For large herbivores living in highly dynamic environments, maintaining range fidelity has the potential to facilitate the exploitation of predictable resources while minimising energy expenditure. We evaluate this expectation by examining how the seasonal range fidelity of African elephants (*Loxodonta africana*) in the Kruger National Park, South Africa is affected by spatiotemporal variation in environmental conditions (vegetation quality, temperature, rainfall, and fire). Eight-years of GPS collar data were used to analyse the similarity in seasonal utilisation distributions for thirteen family groups. Elephants exhibited remarkable consistency in their seasonal range fidelity across the study with rainfall emerging as a key driver of space-use. Within years, high range fidelity from summer to autumn and from autumn to winter was driven by increased rainfall and the retention of high-quality vegetation. Across years, sequential autumn seasons demonstrated the lowest levels of range fidelity due to inter-annual variability in the wet to dry season transition, resulting in unpredictable resource availability. Understanding seasonal space use is important for determining the effects of future variability in environmental conditions on elephant populations, particularly when it comes to management interventions. Indeed, over the coming decades climate change is predicted to drive greater variability in rainfall and elevated temperatures in African savanna ecosystems. The impacts of climate change also present particular challenges for elephants living in fragmented or human-transformed habitats where the opportunity for seasonal range shifts are greatly constrained.

## Introduction

Movement studies across a range of mammal species have shown that animals often return to familiar sites to access predictable resources^1–3^. Maintaining range fidelity enables individuals to maximise nutritional and energetic intake by exploiting the location of known foraging opportunities while minimising the risk of potential threats^2,4,5^. For example, female mule deer (*Odocoileus hemionus*) exhibit high range philopatry during the summer months when they give birth, raise offspring and accrue fat reserves^6^, while site fidelity in southern Australian bottlenose dolphins (*Tursiops* cf. *australis*) has been linked with highly productive habitats and long-lasting social bonds^7^. Determining the extent of range fidelity exhibited by individual animals can provide important insights into how these movement decisions can scale up to influence population dynamics and community level processes^8,9^.

Large herbivores inhabiting seasonally dynamic environments may demonstrate high levels of range fidelity due to the predictability of resources in specific habitats during a given season^10^. This is a common driver of migratory behaviour among species that consistently track resources across the landscape during the spring green-up—referred to as ‘surfing the green wave’^11^. However, exploratory movement is less of a risk to animals occupying relatively homogeneous habitats due to dependability in conditions, and range fidelity is therefore a less common strategy for these species^12^. Similarly, animals inhabiting unpredictable environments are expected to exhibit ‘nomadic’ behaviour with comparatively low consistency in movement behaviour over space and time^11,13^. Ultimately, these movement strategies are shaped by a combination of extrinsic variables that drive resource quality and predictability in the environment and the intrinsic factors related to an individual’s physiology, behavioural plasticity and life history^14,15^.

Fidelity is not just limited to range location, but is also dependent upon variation in range size^1,2^. An individual may change the size of its range while maintaining the core area of habitat use, consequently presenting relatively high range fidelity, but with a change in the degree of range overlap^1^. The nutritional, energetic and reproductive demands of an animal are fundamental in driving range size and overlap^16,17^. However, these are further influenced by a myriad of extrinsic and intrinsic factors that play out over time and space. For large herbivores, these include habitat quality, population density, the abundance of predators, human disturbance, sociality, body size, digestive physiology and life history^1,18,19^.

The movement behaviour of African savanna elephants (*Loxodonta africana*) is predominantly driven by their considerable energetic and nutritional requirements^20,21^, as well as their dependence on surface water availability^22–24^. Elephants are true mixed feeders, demonstrating a seasonal dietary shift from grazing in the summer wet season to browsing in the winter dry season^25,26^. This is because vegetation productivity is higher in the wet season, which is associated with increased nutritional value and digestibility of plant material^25,27,28^. Moreover, an inverse relationship between rainfall and movement autocorrelation has been exhibited, showing that once released from resource limitations in the dry season, elephants become less constrained in their space use due to increased resource availability^29–31^.

By adapting their movements to correspond with seasonal resource availability, elephants are able to minimise the energetic costs associated with moving through the heterogeneous savanna landscape^32–34^, while balancing critical physiological requirements, such as thermoregulation^35^. Fire meanwhile, is a key ecological driver that can shape the structure and species composition of the savanna through the removal of plant biomass, which temporarily reduces primary productivity until a green flush of vegetation is stimulated^36^. Elephant distribution patterns have been shown to be influenced by fire return period, with family groups selecting for areas experiencing lower fire frequency^37^.

Quantifying an individual’s space use over time is crucial for understanding both their consistency in movement behaviour and predictability of the habitats that they occupy^2,38,39^. Through the generation of utilisation distributions (UDs), we are now able to quantify the probability of occurrence, and thus, concentration of space use for an individual across temporal scales^40,41^. However, there are a limited number of studies that have considered the intraspecific similarity in UDs across multiple, recurrent seasons, with fewer still explicitly attempting to identify the key drivers of fidelity in range use (but see^2,10,42^).

Identifying the environmental context in which individuals are driven to make their movement decisions, allows ecologists to make informed predictions about the effects of fluctuations in resource availability or anthropogenic disturbance on large herbivore space use over time, which is crucial in the face of unprecedented and rapid environmental change^6,10,31^. These predictions are vital not only to species conservation, but also in determining the effects that species such as the elephant may have on their ecosystems, including altering habitat structure^43–45^. The aim of this study was to identify the consequences of spatial and temporal resource variability on seasonal range fidelity of African elephant family groups in Kruger National Park, South Africa. The objectives were to determine (1) the overall consistency in seasonal space use within and across multiple years, and (2) identify the key environmental variables driving seasonal range fidelity.

## Methods

### Study site

**Figure 1:**
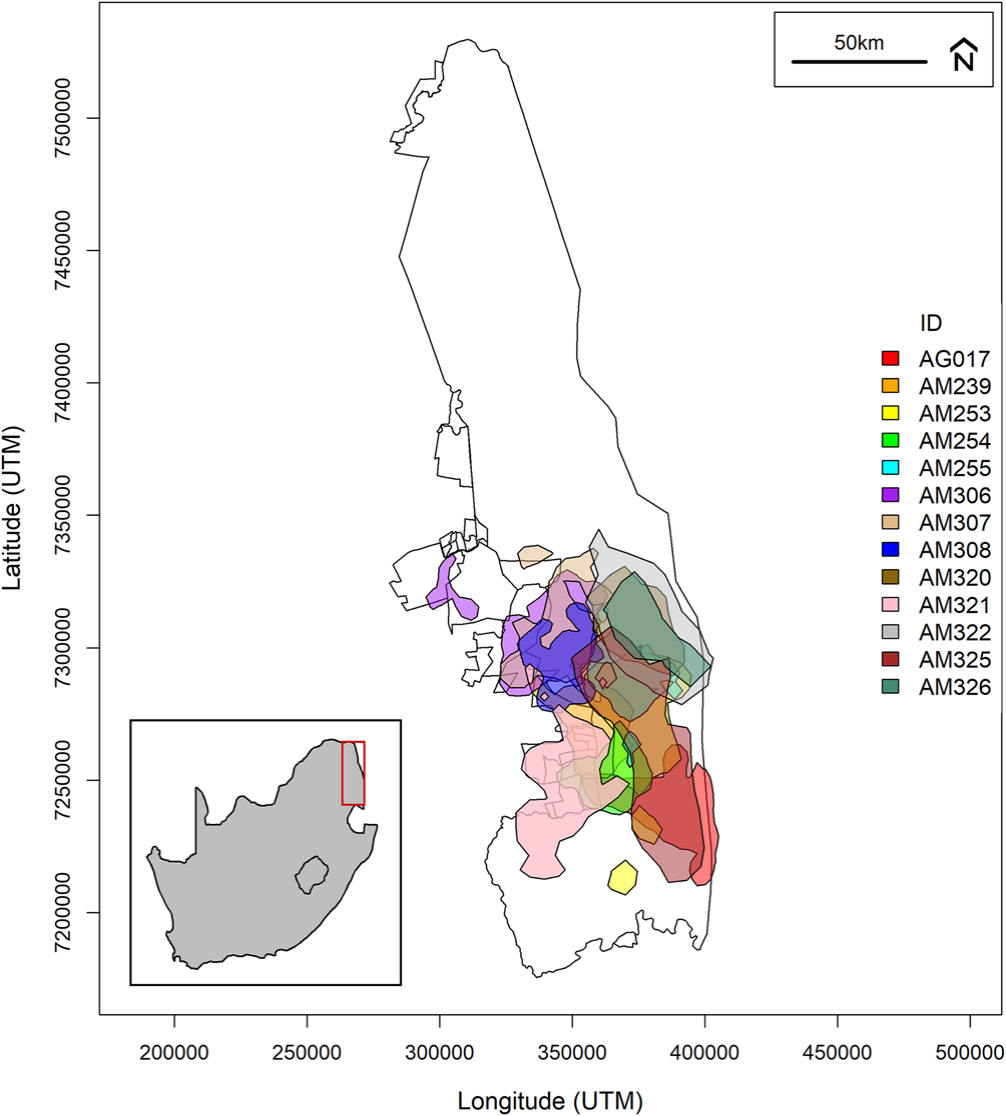
Home ranges (90% Kernel) of all thirteen elephant family groups in KNP and associated private reserves using the data collected over the duration of the study period (see Table S1 for annual and seasonal range size comparisons for each family). The red box on the inset map highlights the location of KNP in South Africa. This map was created in RStudio^52^ using the ‘maps’ package^50^ database, while the coordinates of the study area were projected from the WGS84 datum to UTM zone 36S.

Kruger National Park (KNP) is located in South Africa and extends over 19,485 km^2^ of heterogeneous savanna landscape (Fig. 1). The southern region of KNP receives the most rain, ranging from 500 to 700 mm year^−1^, while the northern section receives approximately 300–500 mm year^−1^^46,47^. The east of KNP is formed of nutrient-rich basaltic soils, with the west comprised of less-fertile granite soils^47^. Northern KNP is dominated by *Mopane* woodlands^47,48^, and in the south, *Combretum* and *Acacia.* There is broad leaf savanna in the west and fine leaf in the east^47^. KNP experiences hotter summer wet seasons with temperatures reaching up to 33 °C, and cooler winter dry seasons with temperatures dropping to around 6 °C^47^. In 2015, the elephant population was estimated to number a minimum of 17,086 individuals, with a growth of 4.2% per annum since 2003^49^.

### Movement data

Location data were collected from 13 female elephants between 2006 and 2013 using Global Positioning Satellite (GPS) Collars (see Fig. 1 and Table S1) (Africa Wildlife Tracking cc., South Africa). The collaring procedure is explained in more detail in Birkett et al.^29^. Each of the collared individuals were selected from a different family group (adult females and their dependent young). Collars recorded locations of the animal every thirty minutes with an average estimated positional dilution of precision (PDOP) value < 2.0^29^ and a maximum circular error probability (CEP) of 4 m. These values are indicative of overall low position estimation error. The methods were approved by the University of KwaZulu-Natal Ethics Committee and conducted in accordance with institutional guidelines.

Utilisation distribution (UD: the probability of animal occurrence in space and time) estimations were calculated using the dynamic Brownian bridge movement model (dBBMM), implemented using the package ‘move’^51^ in Rstudio^52^. The dBBMM builds on the original Brownian bridge movement model (BBMM;^53^) by incorporating both temporal and behavioural characteristics of movement paths into home range estimation^40^. Such features of a movement model are key for estimating the space use of a species like the African elephant, which is known for making highly directional movements over long distances towards known areas of resource abundance^54,55^. After visual data inspection and recommendations by Kranstauber et al.^40^, we specified our moving window size as 31 (equivalent to 15.5 h), with a margin size of 11 (equivalent to 5.5 h). These parameters were based on the number of GPS locations recorded per 24 h for an individual elephant, while also ensuring that sudden changes in movement variance associated with environmental events, such as the increases in temperature, onset of seasonal rainfall and fire could be detected.

### Environmental data

Seasons were classified as summer (December–February), autumn (March–May), winter (June–August) and spring (September–November). These were defined using phenological research into green-up dates^56^ and the analyses of solar radiation in KNP^57^. By using four seasons, we were also able to capture the transitional periods between the traditional wet (spring & summer) and dry (autumn & winter) seasons. Environmental factors considered in this study included green vegetation density, rainfall and mean daily temperature. Datasets for vegetation quality were accessed through the Environmental-Data Automated Track Annotation System (Env-DATA) provided by Movebank ^58^. Summaries of the Moderate Resolution Imaging Spectroradiometer (MODIS) enhanced vegetation index (EVI) (250 × 250 m resolution)—a measure of vegetation greenness—were obtained every 16 days^59^ and the mean value for each seasonal UD was calculated.

SANParks collected monthly rainfall measurements from twenty-three weather stations across KNP enabling rainfall to be interpolated for each season. Average measurements of rainfall were calculated for each seasonal UD. We also included the average rainfall for the preceding season for each UD, due to the influence of rainfall on savanna primary productivity over subsequent weeks and months. These values were assigned to the variable “lag rainfall”. Temperature records were obtained from the South African Weather Service, with the mean daily values calculated for each season. Prescribed burning was conducted in KNP between the years of 2005–2013, across all seasons. Three measurements for fire were calculated for each seasonal UD: the percentage of area burned, percentage of area burned in the last fire before the season started and the time since last fire. Surface water was not included in our analysis, as it is readily available across KNP in the form of rivers, waterholes and pumped dams, and while no doubt a key driver of elephant movement, the relative availability is not expected to vary significantly between seasons^60^.

We predicted that rainfall would have the greatest influence on seasonal range fidelity, with increased levels of rainfall resulting in lower fidelity during the transition from dry to wet season as forage abundance and quality improved^30,33^. Whereas higher rainfall in the transition from wet to dry season would result in greater range fidelity as foraging opportunities were maintained into the winter^61^. Similarly, we anticipated that an increase in primary productivity from the dry to wet season would result in decreased fidelity due to reduced constraints on movement^29,62^. But conditions that led to primary productivity being maintained from summer into winter would lead to higher fidelity as summer ranges continued to provide key forage resources^27^. We also expected lower range fidelity when the second season experiences an increase in temperature, as this is a key driver of vegetation phenology and growth^56^. Fire is more complex to predict, as burn events can cause significant disturbance and displace animals from preferred habitats^63^. However, the post-fire flush of vegetation may also provide important foraging opportunities depending upon the season^37,64^. Finally, it is important to note that elephant ranging behaviour may well be driven by a combination of these environmental variables. We therefore employed an information-theoretic approach to evaluate a set of candidate models and quantify the contribution of each variable in explaining the range fidelity observed across seasonal comparisons.

### Statistical analysis of movement

All statistical analyses were conducted in the R environment for statistical computing. Family group movement tracks were divided into segments (“bursted”) categorised by season (summer, autumn, winter, spring) and year, via the ‘move’ package^51^. Seasonal UDs were calculated for each family group according to their “bursted” seasonal movements, before measuring similarity in UD rasters with the Earth Mover’s Distance (EMD) function^51^. The EMD quantifies the similarity between UDs by calculating a measure of minimal amount of effort taken to shape one UD into another. This provides a spatially explicit comparison between UDs, delivering a more informed comparison than the volume of intersection (VI) or Bhattacharyya’s affinity, because it considers spatial proximity rather than just exclusive overlap^41^.

EMD values were compared across all paired combinations for each family group, including consecutive seasons within the same year (e.g. winter 2008 and spring 2008) and the same seasons across consecutive years (e.g. summer 2008 and summer 2009). EMD values were divided by the threshold value (the largest distance that the united locations of each UD contained), and converted to lie between zero and one, with zero representing two identical UDs.

We fitted generalised linear mixed effects models using the ‘glmmTMB’ package in R^65^ with EMD included as the response variable. Fixed effects included the difference in environmental variables for each season-year pair of UDs. These were calculated by subtracting values of the first season from the second (e.g. spring 2008 rainfall – winter 2008 rainfall). For example, negative values in rainfall difference indicates lower rainfall in the second season compared to the first, and vice versa. The environmental variables included: EVI (vegetation greenness: scale 0–1), mean daily temperature (°C), rainfall (converted from mm to m), lag rainfall (converted from mm to m), percentage of UD burned within season (BIS) (%), percentage of UD burned in last fire before season (BLS) (%), and time since last burn in UD before season (TSLB) (days).

Testing for correlation between fixed effects demonstrated multicollinearity between rainfall, temperature and EVI for consecutive seasonal comparisons within the same year. These fixed effects (with a correlation coefficient greater than 0.4) were excluded from the same models. However, no evidence of collinearity was found between fixed effects when comparing seasonal space use across years. Thirty-three candidate models were generated a priori for EMD for consecutive seasonal comparisons within the same year (Table S2a), and thirty-nine for the same seasonal comparisons between consecutive years (Table S2b). All models included elephant family identity as a random effect to account for repeated observations of the same group. We also included three interactions (season * rainfall, season * temperature and season * EVI) to determine how variation in environmental conditions across seasonal comparisons influenced range fidelity.

Akaike’s Information Criterion adjusted for small sample size (AICc) was used for model selection^66^. Model averaging was conducted on the models that accounted for ≥ 0.95 of the AICc weight using the ‘AICcmodavg’ package^67^. The significance for each environmental variable that featured in the top models was assessed by extracting the β estimates and 95% confidence intervals (CI) and establishing whether these CIs overlapped zero.

### Ethics approval

Ethical approval for the collaring of elephants was obtained from the University of Kwazulu-Natal Animal Ethics sub-committee (Ref. 009/10/Animal). This research was also part of a registered and approved SANParks project, in association with Kruger National Park and Scientific Services (Ref: BIRPJ743).

## Results

**Figure 2:**
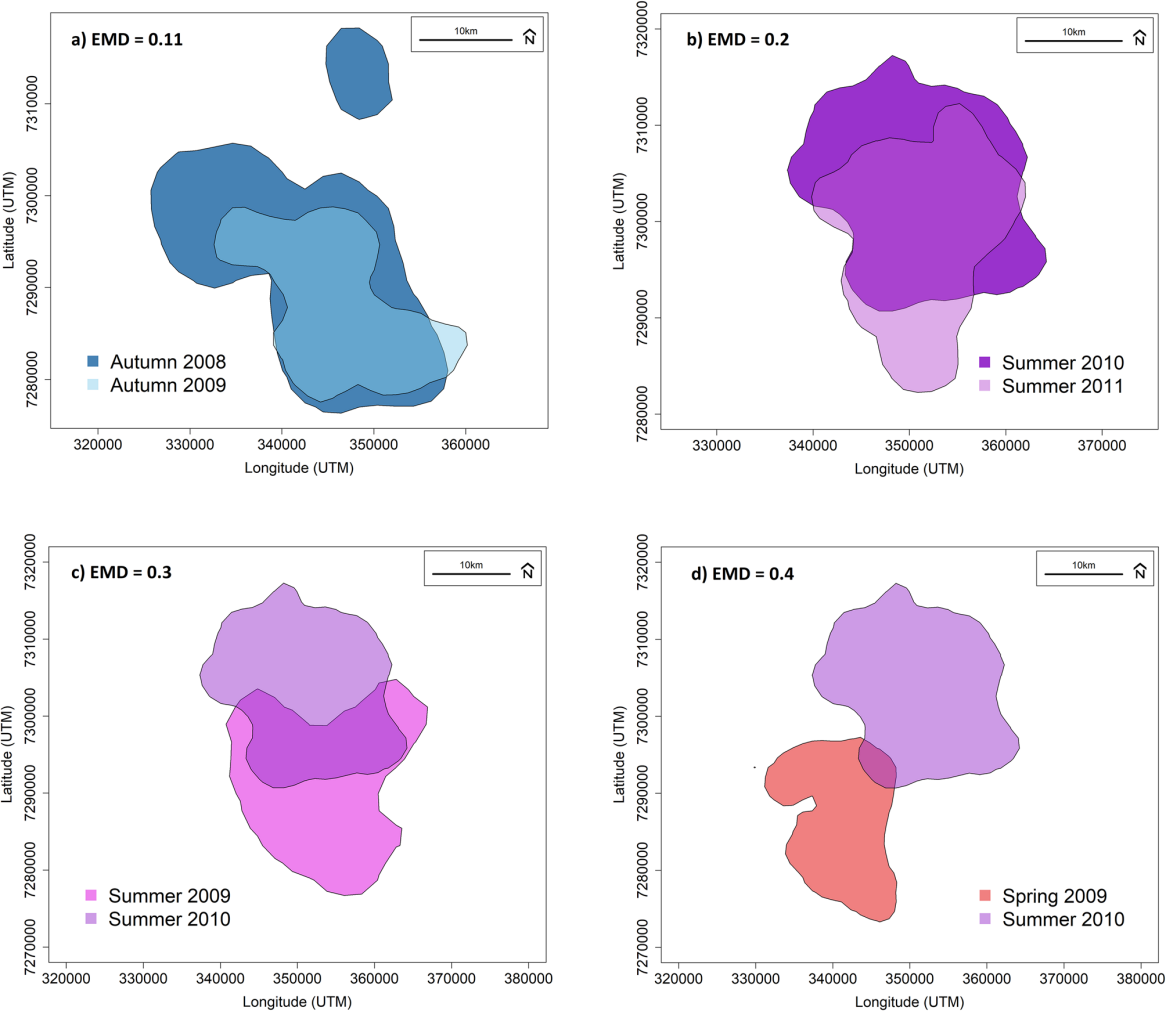
Visualisation of various Earth Mover's Distance (EMD) values for seasonal space use comparisons of family group AM308: (**a**) autumn 2008 and autumn 2009, (**b**) summer 2010 and summer 2011, (**c**) summer 2009 and summer 2010, and (**d**) spring 2009 and summer 2010.

The average EMD value among all family groups was 0.13 (SE ± 0.006) for consecutive seasonal comparisons within the same year and 0.12 (SE ± 0.007) for the same seasonal comparisons across consecutive years (scale is 0–1, with zero representing two identical UDs; see Fig. 2). For within and between year seasonal space use comparisons the EMD values ranged from 0.01 to 0.45 (n = 166) and 0.01–0.39 (n = 135), respectively. Over 80% of seasonal comparisons had an EMD value of 0.2 or less, indicating that the elephants exhibited a high level of seasonal range fidelity within and between years.

### Consecutive seasons within the same year

**Figure 3:**
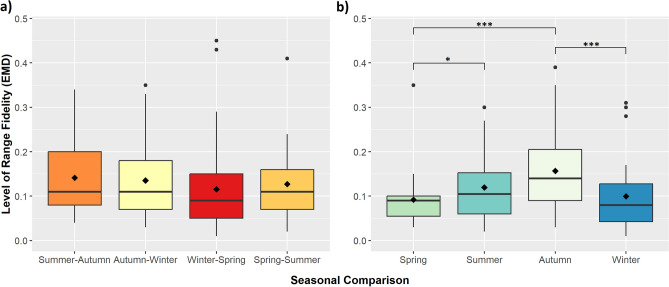
Seasonal space use comparisons of elephants in KNP for (**a**) consecutive seasons within the same year and (**b**) the same seasons between consecutive years. Means are indicated by the ◊ symbol. Significant differences in Earth Mover’s Distance (EMD) values for seasonal space use comparisons across years (significance codes: ‘*’ 0.05 ‘***’ 0.001).

**Figure 4:**
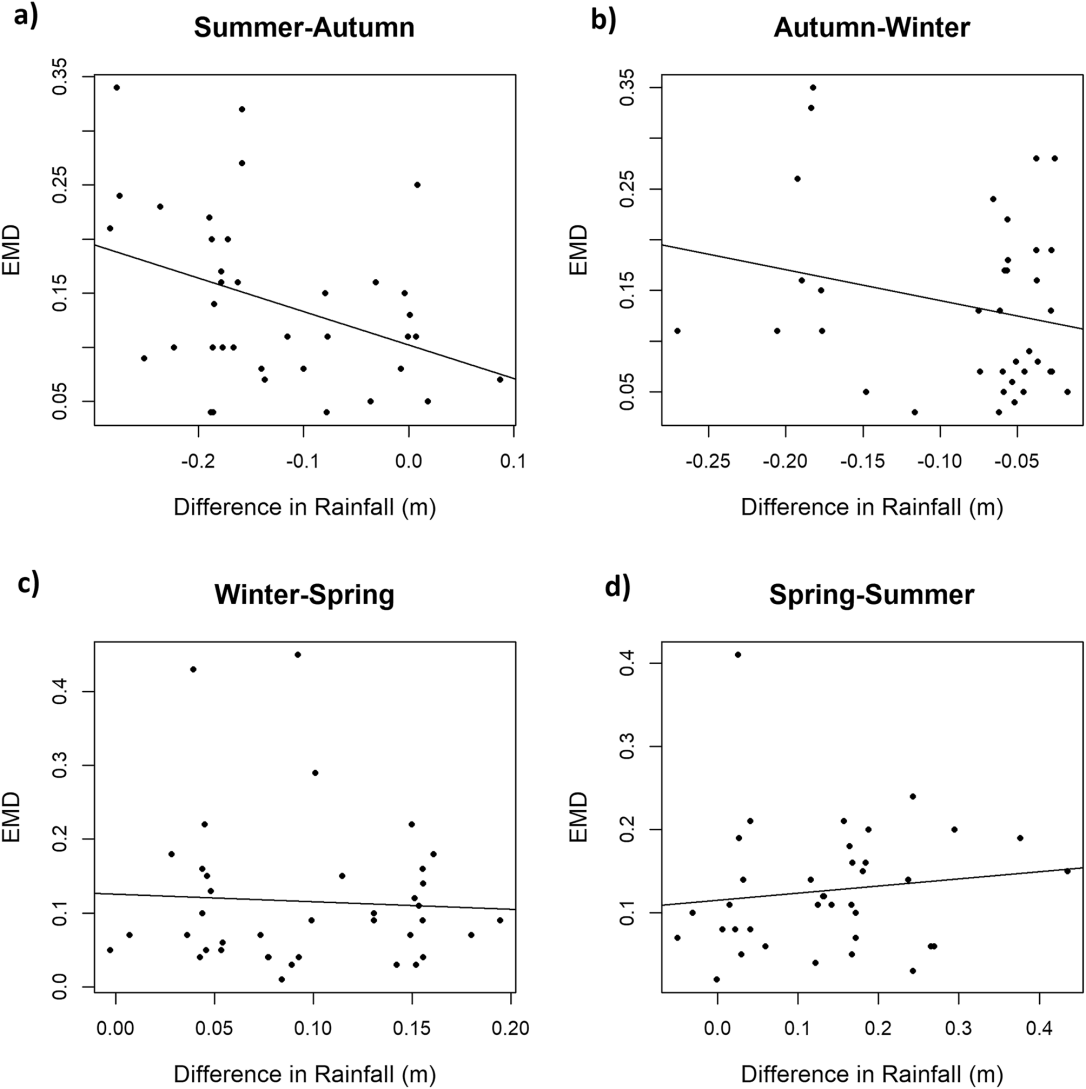
The relationship between difference in rainfall for consecutive seasons within the same year and Earth Mover’s Distance (EMD) for (**a**) summer and autumn (**b**) autumn and winter (**c**) winter and spring and (**d**) spring and summer comparisons. Negative values in rainfall difference indicates lower rainfall in the second season compared to the first, and vice versa. Lower values in EMD indicate higher fidelity.

Mean EMD remained consistently low during these seasonal transitions with no significant difference in range fidelity (Fig. 3a and Table S3). Consistency in space use for consecutive seasons in the same year was best explained by a model that included an interaction between rainfall and season, accounting for 28% of the AICc weight (Table 1). Model averaging indicated that difference in rainfall was the only variable found to be significant in predicting EMD (Table 2). Range fidelity increased when there was greater rainfall in the second season for autumn–winter and summer-autumn comparisons (Fig. 4).

**Table 1:** Top models for consecutive seasonal comparisons within the same year (only models contributing ≥ 0.04 of the AICc weight are shown).

Model	K	ΔAICc	AICc weight
Rain : seasonal comparison	7	0	0.28
Rain	4	1.2	0.15
Rain + BLS	5	2.0	0.10
Temp	4	3.3	0.05
Rain + BIS	5	3.3	0.05
Null	3	3.8	0.04
Rain + BIS + BLS	6	4.1	0.04

For all models, elephant family group identity was included as a random effect. For consecutive seasons within the same year, the interaction between rainfall and specific seasonal comparison was explored.

*Temp* temperature, *BIS* percentage of UD burned within season, *BLS* percentage of UD burned in last fire before season, *TSLB* time since last burn in UD before season.

**Table 2:** The observed relationship between the response variable (EMD) and the model-averaged parameters from each top model (β-estimate ± 95% CI) for consecutive seasons within the same year.

Parameter	β-estimate	(95% CI)
*Intercept* (**AW**)	0.14	(0.11/0.17)
**Rain : AW**	**− 0.03**	**(− 0.05/0.00)**
**Rain : SA**	**− 0.03**	**(− 0.04/− 0.01)**
Rain : SpS	0.01	(− 0.01/0.03)
Rain : WSp	0.00	(− 0.03/0.02)
**Rain**	**− 0.01**	**(− 0.02/0.00)**
BLS	0.00	(0.00/0.00)
Temp	0.00	(0.00/0.00)
BIS	0.00	(0.00/0.00)
TSLB	0.00	(0.00/0.00)
Rain lag	0.04	(− 0.06/0.14)
EVI	− 0.03	(− 0.13/0.07)
SA	0.01	(− 0.03/0.04)
SpS	0.00	(− 0.06/0.07)
WSp	− 0.01	(− 0.08/0.05)

*AW* indicates autumn–winter comparison, *SA* summer-autumn, *SpS* spring–summer and *WSp* winter-spring, *Temp* temperature, *BIS* percentage of UD burned within season, *BLS* percentage of UD burned in last fire before season, *TSLB* time since last burn in UD before season. Intercept taken as AW.

The β-estimates with CI that do not overlap zero are indicated in bold.

### The same seasons between consecutive years

Mean EMD values were highest for autumn comparisons than any other season (spring: EMD = 0.09 ± 0.01; winter: EMD = 0.10 ± 0.01; summer: EMD = 0.12 ± 0.01; autumn: EMD = 0.16 ± 0.02), indicating that elephant family groups showed the least range fidelity from one autumn to the next. Additionally, EMD values were similar for both spring and winter comparisons (Fig. 3b). EMD values in autumn were significantly higher than in both winter (*p* < 0.001) and spring (*p* < 0.001), and significantly higher in summer than in spring (*p* < 0.05) (Fig. 3b and Table S4).

An analysis of EMD for the same season across consecutive years produced eight top models (Table 3). Our top model for explaining consistency in seasonal space use across consecutive years included an additive model for season and time since last burn within range area, accounting for just under a third of the 95% AICc weight (Table 3). Model averaging revealed that EMD values in spring, summer and winter were all significantly different when compared with autumn (Table 4).

**Table 3:** Top models for the same seasonal comparisons between consecutive years (only models contributing ≥0.04 of the AICc weight are shown).

Model	K	ΔAICc	AICc weight
Season + TSLB	7	0.0	0.32
Season	6	0.6	0.24
Season + Rain	7	2.1	0.11
Season + Temp	7	2.5	0.09
Season + EVI	7	2.5	0.09
Season + Rain + Temp	8	4.2	0.04
Season + EVI + Rain	8	4.3	0.04
Season + EVI + Temp	8	4.3	0.04

For all models, elephant family group identity was included as a random effect. *Temp* temperature, *BIS* percentage of UD burned within season, *BLS* percentage of UD burned in last fire before season, *TSLB* time since last burn in UD before season.

**Table 4:** The observed relationship between the response variable (EMD) and the model-averaged parameters from each top model (β-estimate ± 95% CI) for the same seasons between consecutive years.

Parameter	β-estimate	(95% CI)
*Intercept ***(Autumn)**	**0.17**	**(0.13/0.21)**
**Summer**	**− 0.03**	**(− 0.07/0.00)**
**Spring**	**− 0.07**	**(− 0.10/− 0.03)**
**Winter**	**− 0.06**	**(− 0.09 /− 0.03)**
TSLB	0.00	(0.00/0.00)
Rain	0.01	(− 0.01/0.02)
Temp	0.00	(− 0.01/0.01)
EVI	0.07	(− 0.18/0.32)

*Temp* temperature, *BIS* percentage of UD burned within season, *BLS* percentage of UD burned in last fire before season, *TSLB* time since last burn in UD before season. See Table S5 for individual seasonal comparisons.

The β-estimates with CI that do not overlap zero are indicated in bold.

## Discussion

The results from our study indicate high seasonal range fidelity across all thirteen elephant family groups. Indeed, although there were significant differences in seasonal range fidelity across years (particularly during the autumn months), the average EMD values within and between years remained low and remarkably consistent. Given the comparatively long-term duration of our study, these results indicate that the elephants within KNP tend to concentrate their space use in familiar areas, demonstrating high fidelity in both seasonal ranging patterns and spatial use. Through the maintenance of range fidelity, species living in heterogeneous environments are able to maximise their individual fitness by exploiting predictable resources^3,10,68^. Developing an understanding of the conditions that influence similarity in space use is vital for population management, particularly for an ecosystem engineer that can range over hundreds and even thousands of km^2^ but is also sensitive to changes in temporal resource availability^69,70^.

For African elephants, strategically maintaining high range fidelity could minimise energy expenditure through detailed knowledge on predictable resource availability. Certainly, movements have been shown to be strongly shaped by energetic and nutritional considerations at the landscape scale. For example, elephants in northern Kenya avoid comparatively steep slopes despite the availability of abundant forage on higher ground^71^, while elephants in the semi-arid Etosha National Park have been shown to access the closest waterhole 90% of the time, demonstrating a detailed spatial knowledge over large scales that facilitates optimal movement^55^. Moreover, a recent study in KNP revealed facultative shuttling (moving frequently between water and safer sites) towards water sources, with a greater proportion of time spent near water at peak temperatures during the day, especially in the dry season^35^. These results demonstrate how elephants are able to modify their movements in response to thermal stress, whilst utilising their considerable spatial memory to efficiently access key resources^55,62,72^.

It is important to highlight that the consistent availability of surface water in KNP may facilitate the high seasonal range fidelity observed across the elephant family groups in this study. However, a relative abundance of water could also release elephants to range more widely and exploit a variety of habitats compared with arid regions where access to water can constrain movements, especially in the dry season^27^. In support of this, analysis of fine-scale movements of elephants to and from water in KNP revealed comparatively low-level fidelity to specific water points^35^. By essentially accounting for surface water in our study, it was possible to explore consistency in space-use as a function of environmental conditions and forage resource availability that has relevance beyond the study population.

At the group level, this high seasonal fidelity within and across years, will inevitably result in heterogeneity in habitat use within ranges due to variation in foraging intensity and primary productivity^70,73^. It is reasonable to suggest that this may well translate to pronounced variation in elephant activity across the national park, with family groups remaining consistent in their space use and thus concentrating their foraging on vegetation in the core of the range, while other habitats experience lower browsing pressure. The high range fidelity demonstrated in our study could play a pivotal role in enhancing the biodiversity of KNP. Indeed, research has continued to support the ‘habitat heterogeneity hypothesis’, suggesting that heterogeneity increases biodiversity through greater niche provision^74–77^. However, it is worth noting that at the population-level elephant distribution patterns in KNP may have become more homogenous over the past three decades as a function of population growth^37^. From 1985 to 2004 the annual census revealed significant signs of clustering and sexual segregation. During the subsequent decade this was no longer observed and the elephants were distributed throughout KNP habitat types, albeit concentrated closer to major rivers^37^. These annual counts can only provide a relatively coarse snapshot of elephant distribution and further investigation should consider if elephant ranges correspond with threatened habitats, or endemic species that may be unable to adapt to intense browsing pressure^24,78^.

For consecutive seasons within the same year, rainfall was found to be the only significant driver of seasonal range fidelity. During summer to autumn and autumn to winter transitions, family groups maintained site fidelity when rainfall remained comparatively high in the second season. The winter months are typically the driest, while autumn represents the transition from the wet to the dry season and an elephant dietary shift from grass to browse^26,79^. Patterns of higher fidelity following these seasonal transitions is likely due to exploitation of aseasonal rainfall (i.e. unusually wet conditions at a time that is typically dry) and the subsequent retention of higher quality vegetation, such as grasses in the autumn and palatable deciduous trees in the winter, which has been shown to influence ungulate population dynamics^80,81^.

Interestingly, Thaker et al.^35^ revealed that the daily distance moved by elephants in KNP was consistent across both wet and dry seasons, which was attributed to high density and accessibility of waterholes and surface water in the south of KNP. Given that the elephants are largely range faithful, it is possible that, with a continuation of rainfall into the dry season, elephants are less inclined to adjust their seasonal ranges due to familiarity with the locations and availability of key resources^54,55^. Indeed, elephants appear to be very effective at integrating long term information on environmental conditions into their movement strategies^34^.

The lowest levels of seasonal range fidelity between years occurred in the autumn months, while EMD values for winter, spring and summer indicated higher fidelity and greater associated familiarity with spatiotemporal resource availability. As demonstrated by Birkett et al.^29^, and supported by our intra-annual analyses, the breakpoints at which seasonal transitions occur are subject to considerable variation across years. This variability in EMD during the autumn months is indicative of the unpredictability in location and longevity of resources moving into the dry season^56^. Seasonal dietary shift is a primary adaptation of elephants to fluctuations in resource availability, with grass predominantly being consumed in the wet season and an increasing dependence on woody browse during the winter months^25,26,82^. Reliance on these resources continues into the spring until elephants are released from the dry season and the first green-up of vegetation occurs^26,83^. Climate change is likely to amplify variation in the timing of key phenological processes^84^, which may have significant effects on elephant movement. This is highlighted by the significant difference in fidelity across consecutive years between spring and summer, possibly attributable to the previous early flush of some deciduous trees in spring and an early release in constrained movements, even without rain.

The elephants in KNP did not significantly alter their space use to either avoid or utilise burn patches within their seasonal ranges. However, previous studies demonstrating that elephants initially avoid burned areas in the short term and return once the vegetation recovers^37,63^. It is therefore possible that our temporal scale for analysis was too coarse to highlight the short-term effects of fire on movement. Similarly, change in temperature was not highlighted as a key predictor of range fidelity. Indeed, research has found that elephants in KNP respond to “landscape of thermal stress” at finer temporal scales by periodically shuttling to water during the hottest times of the day^35^.

Research comparing elephant space use has largely focussed on calculating areas of explicit spatial overlap^31,85^. However, this approach does not account for the proximity in space use between seasonal distributions, resulting in space use similarity being oversimplified. The Earth mover’s distance used in our study has enabled the integration of spatially explicit comparisons of seasonal range use alongside accurate depictions of environmental variability. We have highlighted that rainfall has a significant influence on range fidelity during the dry season. Notably, rainfall in the previous season was not found to be a significant driver of fidelity, which suggests that the elephants in KNP are responding to current forage availability, rather than longer-term changes in plant phenology. However, as rapid advances in technology are enabling ever more detailed movement data to be collected and analysed^86,87^, there is the potential for future studies to explore range shifts that track the measurable effects of climate change across multiple years and populations. Likewise, in this study we only considered the resource availability and environmental conditions within the area of the UD. An important next step is to incorporate elephant perception of key ecological and environmental drivers that lie beyond the boundaries of the range, but which likely shape movement decisions, nonetheless.

Although no detailed demographic information relating to the composition of family groups within this study is available, a recent study demonstrated that range shifts and expansions of elephants in northern Kenya were predominantly attributed to generational turnover, with loss of mature adults significant in predicting increase in range size and centroid shift^62^. Family groups also demonstrated a clear shift in movement patterns away from increased poaching levels, and towards areas of greater primary productivity^62^. These results highlight the role of both environmental and anthropogenic drivers on intraspecific philopatry. However, within KNP, the capacity for anthropogenic disturbance is limited (beyond tourist activity in rest camps and along park roads). Therefore, a strong focus on the effects of environmental variation on elephant movement patterns remains key to future management plans and biodiversity conservation within the park.

Understanding the drivers of elephant space use is useful in predicting movement patterns that are likely to result from the longer-term effects of climate change^88,89^, particularly given they are keystone species capable of shaping habitat structure and ecosystem function^44,90,91^. Specifically, it is predicted that climate change will lead to increased variability in rainfall patterns and higher temperatures in African savannas^92,93^. The elephants in KNP have demonstrated their ability to detect and respond to aseasonal rainfall patterns by adjusting their ranging behaviour. Dry seasons that experience below average rainfall are likely to drive reduced range fidelity as elephants seek out remaining foraging opportunities, a situation which could become exacerbated with increased drought frequency. Ultimately, the altered movement and foraging behaviour of elephants at the population-level could have cascading effects on vegetation^45,94^ other herbivore species^95^, and predators^96,97^.

Identifying areas of concentrated elephant space use allows scientists and managers to highlight potential areas of human-wildlife conflict and habitats that are vulnerable to sustained grazing and browsing pressure. Indeed, such highly concentrated utilisation may shape biodiversity, especially if these areas overlap with those of range-restricted species. Such predictions are also very useful in assisting with the sustainable management of elephant populations, including the preservation of landscape connectivity and vital movement corridors^98–100^. We have demonstrated that rainfall is the overriding factor influencing elephant range fidelity in KNP where surface water availability is abundant and human disturbance comparatively low. This has considerable implications for elephant conservation as the effects of climate change accelerate and environmental conditions become less predictable across an already fragmented species range.

## Supplementary Information

Supplementary Tables.

## Data Availability

Data are archived on the Movebank (movebank.org) website: “African Elephant Slotow Kruger”.
