# Supplementary material for: Seasonal range fidelity of a megaherbivore in response to environmental change
Source: Sci Rep. 2022 Dec 22;12:22008. doi: 10.1038/s41598-022-25334-8 (PMC9780231; doi:10.1038/s41598-022-25334-8)
Supplement: Supplementary file 1 — Supplementary Tables. [file 41598_2022_25334_MOESM1_ESM.docx]

**Supplementary Information**

**Table S1.** Mean (± SE) range sizes across annual and seasonal scales for all 13 family groups.

| ***ID*** | ***Annual range size estimation*** | | | ***Spring range size estimation*** | | | ***Summer range size estimation*** | | | ***Autumn range size estimation*** | | | ***Winter range size estimation*** | | |
| --- | --- | --- | --- | --- | --- | --- | --- | --- | --- | --- | --- | --- | --- | --- | --- |
|  | *Mean (km^2^)* | SE *(km^2^)* | *n* | *Mean (km^2^)* | SE *(km^2^)* | *n* | *Mean (km^2^)* | SE *(km^2^)* | *n* | *Mean (km^2^)* | SE *(km^2^)* | *n* | *Mean (km^2^)* | SE *(km^2^)* | *n* |
| AG017 |  |  |  | 187.5 |  | 1 |  |  |  | 564.1 |  | 1 | 222.7 |  | 1 |
| AM239 | 336.2 | ± 69.8 | 4 | 145.2 | ± 14.8 | 4 | 99.9 | ± 23.3 | 5 | 118.7 | ± 36.7 | 4 | 115.0 | ± 15.4 | 5 |
| AM253 | 231.1 |  | 1 | 113.3 | ± 1.3 | 2 | 92.4 |  | 1 | 37.8 |  | 1 | 66.6 | 29.9 | 2 |
| AM254 | 427.92 |  | 1 | 106.5 | ± 11.8 | 3 | 128.5 | ± 35.7 | 2 | 153.5 | ± 19.8 | 2 | 155.5 | ± 28.1 | 3 |
| AM255 |  |  | 0 | 175.2 |  | 1 |  |  | 0 | 95.1 |  | 1 | 134.4 | ± 47.8 | 2 |
| AM306 | 565.2 | ± 116.6 | 2 | 153.8 | ± 22.2 | 3 | 324.7 | ± 10.4 | 3 | 226.7 | ± 13.7 | 3 | 137.3 | ± 49.5 | 3 |
| AM307 | 329.9 | ± 18.0 | 2 | 109.7 | ± 6.2 | 3 | 169.5 | ± 38.1 | 3 | 155.0 | ± 23.7 | 4 | 85.2 | ± 14.7 | 4 |
| AM308 | 427.2 | ± 46.4 | 2 | 123.5 | ± 16.7 | 3 | 188.3 | ± 22.6 | 3 | 176.2 | ± 19.8 | 4 | 103.1 | ± 19.7 | 4 |
| AM320 | 354.6 | ± 29.8 | 5 | 146.9 | ± 34.6 | 6 | 149.9 | ± 14.1 | 6 | 187.5 | ± 22.3 | 7 | 151.0 | ± 14.3 | 7 |
| AM321 | 434.2 | ± 79.8 | 6 | 209.1 | ± 36.5 | 7 | 311.4 | ± 66.1 | 7 | 167.3 | ± 31.1 | 8 | 115.1 | ± 19.5 | 7 |
| AM322 | 371.4 | ± 63.1 | 3 | 199.4 | ± 64.7 | 3 | 157.9 | ± 17.3 | 4 | 188.9 | ± 29.7 | 4 | 148.2 | ± 22.3 | 2 |
| AM325 | 511.5 | ± 73.8 | 2 | 155.3 | ± 17.8 | 3 | 329.2 | ± 76.8 | 2 | 111.0 | ± 27.0 | 2 | 151.8 | ± 42.2 | 3 |
| AM326 | 453.1 | 23.6 | 7 | 214.7 | ± 32.9 | 6 | 253.0 | ± 24.4 | 7 | 249.3 | ± 20.1 | 8 | 155.1 | ± 8.3 | 6 |

**Table S2.** Structure of candidate models assessed for the Earth Mover’s Distance response variable. a) Consecutive seasonal comparisons within the same year, and b) the same seasonal comparisons between consecutive years. For all models, elephant family group identity was included as a random effect. EVI = Enhanced Vegetation Index (greenness), BIS = percentage of UD burned within season, BLS = percentage of UD burned in last fire before season, TSLB = time since last burn in UD before season.

| 1. *Candidate models for consecutive seasonal comparisons within the same year* |
| --- |
| Null |
| EVI |
| Rain |
| Rain lag |
| Temperature |
| TSLB |
| BLS |
| BIS |
| Seasonal comparison |
| Seasonal comparison * Rain |
| Seasonal comparison * EVI |
| Seasonal comparison * Temperature |
| Seasonal comparison + Rain |
| Seasonal comparison + EVI |
| Seasonal comparison + Temperature |
| EVI + BLS |
| EVI + BIS |
| EVI + TSLB |
| Rain + BIS |
| Rain + BLS |
| Rain lag + BLS |
| Rain lag + TSLB |
| Rain lag + EVI |
| Temperature + BIS |
| Temperature + BLS |
| Temperature + Rain lag |
| EVI + BLS + BIS |
| EVI + Rain lag + TSLB |
| Rain + BIS + BLS |
| Temperature + BIS + BLS |
| Temperature + Rain lag + TSLB |
| EVI + Rain lag + BLS + BIS |
| Temperature + Rain lag + BIS + BLS |

* denotes an interaction

|  |
| --- |
| 1. *Candidate models for the same seasonal comparisons between consecutive years* |
| Null |
| EVI |
| Rain |
| Rain lag |
| Temperature |
| TSLB |
| BLS |
| BIS |
| Season |
| Season * EVI |
| Season * Rain |
| Season * Temperature |
| Season + EVI |
| Season + Rain |
| Season + Temperature |
| Season + TSLB |
| EVI + Rain |
| EVI + Temperature |
| EVI + BLS |
| EVI + BIS |
| EVI + TSLB |
| Rain + BIS |
| Rain + BLS |
| Rain lag + BLS |
| Rain lag + TSLB |
| Rain lag + BIS |
| Rain lag + EVI |
| Temperature + BIS |
| Temperature + BLS |
| Rain + Temperature |
| EVI + BLS + BIS |
| Temperature + BIS + BLS |
| EVI + Rain + Temperature |
| Season + EVI + Rain |
| Season + EVI + Temperature |
| Season + Rain + Temperature |
| Rain + BIS + BLS |
| Season + EVI + Rain + Temperature |
| EVI + Rain + Temperature + BLS + BIS |
| * denotes an interaction |
|  |
|  |
|  |

**Supplementary: GLMM output - seasons**

**Table S3.** Estimated regression parameters, standard errors, *z*-values and *P*-values when exploring the relationship between EMD and season, for consecutive seasons within the same year. Autumn-Winter comparison is the intercept.

|  | Estimate | Std. error | *z* value | *P-*value |
| --- | --- | --- | --- | --- |
| Intercept (Autumn-Winter) | 1.373e-01 | 1.623e-02 | 8.404 | <2e-16 |
| Summer-Autumn | 5.285e-03 | 1.800e-02 | 0.291 | 0.771 |
| Spring-Summer | 4,267e-03 | 3.292e-02 | 0.129 | 0.898 |
| Winter-Spring | -1.260e-02 | 3.398e-02 | 0.368 | 0.713 |

**Table S4.** Estimated regression parameters, standard errors, *z*-values and *P*-values when exploring the relationship between EMD and season, for the same seasons between consecutive years, relevelled with Spring as the intercept.

|  | Estimate | Std. error | *z* value | *P-*value |
| --- | --- | --- | --- | --- |
| Intercept (Spring) | 0.102 | 0.019 | 5.389 | 7.1e-08 |
| Winter | 0.008 | 0.017 | 0.445 | 0.6564 |
| Summer | 0.035 | 0.017 | 2.028 | 0.0425 |
| Autumn | 0.069 | 0.018 | 3.755 | 0.0002 |

**Supplementary: Observed relationships between seasonal comparisons**

**Table S5.** Observed relations between seasonal comparisons following relevelling (β-estimate ±95% CI). The β-estimates with CI that do not overlap zero are indicated in bold.

| Baseline (intercept) | Parameter | β-estimate | (95% CI) |
| --- | --- | --- | --- |
| **Autumn** | **Summer** | **-0.03** | **(-0.07 / 0.00)** |
| **Autumn** | **Spring** | **-0.07** | **(-0.11 / -0.03)** |
| **Autumn** | **Winter** | **-0.06** | **(-0.10 / -0.03)** |
| Winter | Spring | -0.01 | (-0.04 / 0.03) |
| Winter | Summer | 0.03 | (-0.00 / 0.07) |
| **Winter** | **Autumn** | **0.06** | **(0.03 / 0.10)** |
| **Spring** | **Summer** | **0.04** | **(0.00 / 0.07)** |
| **Spring** | **Autumn** | **0.07** | **(0.03 / 0.11)** |
| Spring | Winter | 0.01 | (-0.03 / 0.04) |
| Summer | Autumn | 0.03 | (-0.00 / 0.07) |
| **Summer** | **Winter** | **-0.03** | **(-0.06 / 0.00)** |
| **Summer** | **Spring** | **-0.04** | **(-0.07 / -0.00)** |
|  |  |  |  |
